# Caregivers’ Attitudes towards HIV Testing and Disclosure of HIV Status to At-Risk Children in Rural Uganda

**DOI:** 10.1371/journal.pone.0148950

**Published:** 2016-02-16

**Authors:** Rick Lorenz, Eisha Grant, Winnie Muyindike, Samuel Maling, Claire Card, Carol Henry, Adil J. Nazarali

**Affiliations:** 1 College of Pharmacy and Nutrition, University of Saskatchewan, Saskatoon, Saskatchewan, Canada; 2 Ministry of Health, Kampala, Uganda; 3 Faculty of Medicine, Mbarara University of Science and Technology (MUST), Mbarara, Uganda; 4 Western College of Veterinary Medicine, University of Saskatchewan, Saskatoon, Saskatchewan, Canada; Brown University, UNITED STATES

## Abstract

Caregivers of HIV-positive children were interviewed in the Mbarara and Isingiro districts of Uganda to identify current trends in practices related to HIV testing and the disclosure of HIV status to the child. A total of 28 caregivers of at least one HIV-positive child participated in semi-structured interviews exploring when and why they tested the child for HIV, when the child was informed of their positive status, and what the caregiver did to prepare themselves and the child for status disclosure. For a majority (96%) of respondents, the decision to test the child for HIV was due to existing illness in either the child or a relative. Other common themes identified included the existence of stigma in the caregivers’ communities and doubt that the children truly understood what was being explained to them when their status was disclosed. Most (65%) children were informed of their HIV status between the ages of 5 and 9, with the mean age of disclosure occurring at the age of 7. General provision of HIV information typically began at the same age as disclosure, and as many as two thirds (64%) of the caregivers sought advice from an HIV counsellor prior to disclosure. How a caregiver chose to prepare themselves and the child did not affect the caregiver’s perception of whether the disclosure experience was beneficial or not. These findings suggest that the HIV disclosure experience in Mbarara and Isingiro districts differs from current guidelines, especially with respect to age of disclosure, how caregivers prepare themselves and the child, and approaching disclosure as an ongoing process. The doubts expressed by caregivers regarding the child’s level of HIV understanding following the disclosure experience suggest the children may be insufficiently prepared at the time of the initial disclosure event. The findings also suggest that examining the content of pre-disclosure counselling and HIV education, and how health care professionals are trained to facilitate the disclosure process as important avenues for further research.

## Introduction

As the global HIV epidemic enters its fourth decade, significant advances have been made in HIV prevention and treatment in the developing world. While sub-Saharan Africa remains the hardest-hit region (accounting for the majority of HIV-related deaths, new HIV infections, and greatest HIV prevalence worldwide), the outlook for the area is beginning to show signs of improvement [1]. Improved HIV education and increased access to antenatal care have been credited with reducing the number of new infections reported annually, and increasing access to antiretroviral drug therapies have reduced the number of HIV-related deaths [2]. As programs concerned with expanding the accessibility of vital medication and education to low-income, high-burden areas continue apace, new challenges to HIV-related health promotion continue to present themselves.

Uganda is in the unique position to be an effective prototype for other Sub-Saharan African countries since it has one of the most mature HIV epidemics worldwide, and is one of the first countries to stabilize its HIV epidemic [3]. Currently, Uganda is home to an estimated 1.2 million people living with HIV (including 150,000 children), an estimated 1.2 million orphans due to HIV, and a national HIV prevalence among adults aged 15 to 49 of 7.2% [4]. By contrast, the hardest-hit of the Sub-Saharan African nations can have HIV prevalence rates as high as 20%, with significantly higher rates of HIV incidence, mother to child transmission, and child mortality due to AIDS [1]. For this reason, the challenges encountered by Uganda today may likely be encountered in the future by other nations as their own HIV epidemics stabilize. Likewise, research into HIV management and counselling strategies in Uganda may yield data that prove useful in future health policy development in other nations. The Mbarara and Isingiro districts in western Uganda are particularly attractive sites in which to conduct HIV research: their HIV prevalence has historically been greater than the nation overall and has an HIV prevalence most recently estimated at 8.0% [5] and they possesses a variety of both remote rural and municipal settlements.

One notable challenge involves the increased proportion of HIV infected children who live through school age and into adolescence due to increased access to life-saving drug therapies [6]. As these children mature, the question of when and how they should be made aware of their diagnosis is raised. Worldwide, the majority of HIV-positive children are unaware of their HIV status [7]. Often, the child has never been tested for HIV due to a lack of access to testing services or reluctance on the part of caregivers to get the child tested; indeed, current research suggests that those who are most likely to benefit from HIV testing services and drug therapies, especially in rural or low-income areas, are typically not receiving them [8]. A recent Ugandan study has suggested that, for the majority of caregivers of HIV-positive children, the decision to test the child for HIV occurs only after the child begins exhibiting symptoms of illness [9]. The elimination of mother-to-child transmission of HIV by 2015 is a target as part of the Global AIDS Response Progress report, and in Uganda a reduction in annual HIV incidence among children from 27,660 to 9,629 has been observed between 2011 and 2013 [5]. It has been estimated that 94% of expectant mothers attending antenatal clinics received counselling and testing for HIV in 2011 [10]. This high level of coverage implies knowledge about mother-to-child transmission is high in Uganda. In spite of this, recent efforts to scale-up the elimination of mother-to-child transmission resulted in only 71.7% of positive mothers and only 36.7% of infants receiving ARV’s [5]. It is estimated that 20% of new HIV infections in Uganda are due to mother-to-child transmission, although this may be significantly higher since many births in Uganda occur in rural areas outside of healthcare centres [10]. Even if mother-to-child transmission of HIV were eliminated entirely in Uganda by 2015, the Ministry of Health (Uganda) projections indicate as many as 176,948 children under 15 with HIV would remain [5] and would require appropriate disclosure of their HIV status.

For those that have been tested, many HIV-positive children are not informed of their status [11–13] and are ultimately unaware of their HIV status even if currently receiving treatment [14]. A recent study in Southwestern Uganda suggests that only 31% of children aged 5–17 have been informed of their HIV status, while nearly half of the children are completely unaware of their condition [15]. HIV status knowledge has implications with respect to drug compliance, health behaviours, and the child’s overall prognosis [16, 17], but many hospitals in Uganda currently lack formal policies and procedures for disclosing a positive HIV diagnosis to a child [9, 15].

To date, the majority of research into the disclosure of a diagnosis of a potentially fatal disease has involved chronic diseases other than HIV [18–20], and many of the HIV disclosure guidelines that currently exist are derived from such work [21]. These guidelines recommend HIV counsellors and other health care professionals work with the caregiver to develop a ‘disclosure plan’ that takes into account the needs and goals of the caregiver, the child, and the rest of the family [21]. The most recent guidelines published by the World Health Organisation (WHO) recommend that children of school age have their positive status disclosed to them, with younger children being informed incrementally as they mature; however, the quality of evidence upon which these recommendations are based is noted to be ‘low’ [13, 22]. This recommendation is based on research drawing on Piagetian understanding of cognitive development, which supports the concept of increasing the sophistication of dialogue as the child’s understanding of illness increases [16, 17]. More recently, a formal disclosure framework based on the child’s stage cognitive development has been proposed in the United States [23].

Factors including the age of the child at the time of disclosure, method of disclosure, and involvement of health care professionals or counsellors could conceivably affect whether the disclosure process is beneficial and empowering or traumatic [13, 14, 23, 24].

Although it seems logical that the manner by which an HIV-positive child learns his or her HIV status would have significant repercussions on the child’s psychological state, medication compliance, and overall well-being, minimal research into this question has been conducted in the developing world. Research performed in Sub-Saharan Africa suggests that disclosure of HIV status is often viewed as a discrete event, as opposed to a process [25, 26], but to date few studies in Uganda or elsewhere in sub-Saharan Africa have examined how caregivers choose to prepare themselves and the child prior to disclosure. The Uganda Ministry of Health’s most recently published guidelines recommend that counsellors determine whether the caregiver is willing to discuss HIV and the test results before counselling is initiated, and that counsellors work with the caregivers of an HIV-positive child to plan for the child’s future care [27)].

Studies in industrialized countries examining the effects of pediatric HIV status disclosure suggest that disclosure is associated with improved outcomes when compared with non-disclosure, including benefits to self-esteem and social behaviour [28, 29], but the evidence to date in sub-Saharan Africa remains statistically insignificant [27] and highly subjective [29, 30]. Other research has examined the many potential barriers to disclosure for caregivers of HIV-positive children: focus groups of primary caregivers in South Africa identified stigma, lack of knowledge and communication skills related to HIV, and emotional unpreparedness as factors leading to an overall lack of comfort discussing HIV with the positive children under their care [11]. The fear of stigma, in particular, is often cited as a major barrier to disclosure of a child’s HIV status. As the majority of HIV-positive children were infected perinatally, biological parents of HIV-positive children have reported concern that their child would blame them [11, 31]. Similarly, many parents of HIV-positive children are reluctant to disclose the diagnosis to the child in order to protect them from the stress of keeping such a secret from the community and to spare them the emotional trauma of knowing that they are suffering from a terminal disease [12]. Partial disclosure describes situations where the child is made aware that they are sick, but are unaware that they are HIV positive. This form of disclosure may occur as a result of the child becoming suspicious of why they are being given medication, or it may come as a result of active deception on the part of the caregiver. In either case, disclosure of this nature has been linked with lower adherence to HIV drug regimens as well as increased anxiety and mistrust in the caregiver-child relationship [29].

Evidence from studies conducted in the developed world indicate that children who are aware of their positive status fare better in terms of medication compliance, coping mechanisms, and overall well-being than those children who are unaware [14, 31, 32]; however, there is a lack of evidence in sub-Saharan Africa to determine optimal timing and methodology of HIV disclosure. Just as importantly, there has been little research into how the disclosure process typically unfolds in Uganda and how effective it is in the eyes of stakeholders. Thus, the aim of this study was to interview primary caregivers of HIV-positive children to identify trends related to the age disclosure occurs, how caregivers prepare themselves and the child for the disclosure process, exactly how this process occurs, and the challenges faced during the testing and disclosure process. By characterizing what current practice looks like in this patient population and the results it yields, we hope to obtain a better understanding of what standard practice would entail.

## Methods and Study Design

This study was approved by the University of Saskatchewan’s Behavioural Research Ethics Board, the Mbarara University Institutional Research Ethics Committee (MUST-IREC) and the Uganda National Council for Science and Technology. The study evaluated qualitative data collected from interviews with caregivers of HIV- positive children from two distinct sites. All caregivers presenting for routine visits at the Mbarara Immune Suppression Syndrome (ISS) clinic at Mbarara University of Science and Technology (MUST) were screened against the inclusion criteria by nursing staff. Each eligible caregiver was approached in the waiting area and invited to participate in an interview to be conducted in a private room within the ISS clinic. In rural parishes of Isingiro district, beneficiaries of the Foundation for AIDS Orphaned Children (FAOC) were contacted by FAOC staff at monthly parish meetings; the study was announced verbally in English, Runyankole and Luganda to attendees of the meetings and those wishing to volunteer for an interview to be conducted at their home or another suitably private site were permitted to do so anonymously.

Although the original intent was for participants at both sites to be randomly selected from a pool of all eligible participants, the number of eligible participants was sufficiently low to allow for all to be interviewed. Inclusion criteria were as follows: (i) primary caregiver of at least one HIV-positive child (age 0–14); (ii) caregiver acknowledges that child has been informed of his or her HIV status; and (iii) at least one child is receiving antiretroviral therapy or prophylactic antibiotic therapy. The second criteria also served two purposes; it ensured that participating caregivers had experienced the disclosure experience at least once, while enabling caregivers of multiple children to discuss the experiences they had with children who may not know their status. Written informed consent was obtained in English, Runyankole, or Luganda as appropriate. In situations where the participant’s literacy level prevented written informed consent from being obtained, an equivalent verbal consent agreement was made available. The Ethics committees at both institutions as well as the Uganda National Council for Science and Technology approved these consent procedures. The reading of the form was recorded, and interviewees providing consent were asked to verbally assent to continuing the interview as well as indicating on the consent sheet using either a signature or thumbprint. Participants were informed that their involvement was entirely voluntary and that they may choose to not answer questions if uncomfortable, and may stop the interview at any time. A translator fluent in English, Runyankole, and Luganda and specifically trained to assist in administering the interview questionnaire was present for interviews where the volunteer was unable to conduct the interview in its entirely in English. Using mock interviews, translators were trained to ensure complete fidelity to what was actually said by the interviewer and interviewees without paraphrasing or embellishment, consistency between interviews, and signed a confidentiality agreement prior to assisting in the study.

The interviews focused on the caregiver’s experiences and attitudes regarding the experiences of HIV status testing and the disclosure of the status to the child. The interview format consisted of both open-ended questions where caregivers were instructed to discuss their experiences and challenges as well as discrete, closed-ended questions that aimed to collect numeric and categorical data. All closed-ended questions were followed up with open-ended questions where caregivers were encouraged to describe in their own words the reasoning and context of behind their responses. Interviews were semi-structured and took 45–75 minutes to complete. The transcribed, coded interviews are available to interested researchers upon request from the authors or the University of Saskatchewan’s Behavioural Research Ethics Board. To maintain confidentiality names of participants will be removed prior to sharing of this data. Researchers can send requests to access this data to A.J. Nazarali at aj.nazarali@usask.ca or R. Lorenz at rfl978@mail.usask.ca

All interviews were transcribed by a second translator, who was instructed to look for any discrepancies between what was said by interviewees and what was reported by the primary translator (where applicable). The second translator separately listened to the audio recordings, which contained both the Ugandan questions and answers as well as the first translator’s real-time English translation. Text files of all interview transcripts were imported into HyperResearch 3.5.2. Responses to open-ended questions were used, both individually and collectively, to generate word counts. Frequently used words were examined for context and used to generate the code book. These codes were then applied to transcripts in order to identify emergent themes in the responses.

## Results

A total of 28 caregivers were interviewed (Table 1), of these 21 were the biological parent of the child and 19 of whom self-identified as being HIV positive. Disclosure experiences of 31 children were discussed during the course of the study.

**Table 1:** Characteristics of Caregivers and Children.

Characteristic	Frequency	%^a^	Characteristic	Frequency	%^a^
Age of Caregiver (Years)			Occupation of Caregiver		
19	2	7	Owns business	4	14
21–30	5	18	Labourer	6	21
31–40	12	43	Farmer	10	34
41–50	7	25	Hawker	2	4
51–60	1	4	Mechanic	1	3
60+	1	4	Hairdresser	1	3
**Caregivers**			Tailor	1	3
Male	5	18	Works in shop	1	3
Female	23	82	Cook	1	3
**Marital Status of Caregiver**			None	2	7
Married	12	43	**Place of Dwelling**		
Divorced	7	25	Urban	2	7
Widowed	5	18	Small town	4	14
Never married	4	14	Village	22	79
**HIV Status of Caregiver**			**People in Household**		
Positive	21	75	2–4	7	25
Negative	5	18	5–7	17	61
Not tested	1	4	8 +	4	14
Declined to say	1	4	**Child's Age (years)**		
**Relationship to Child**			0–5	3	10
Biological parent	21	68	6–10	14	45
Step parent	5	16	11–14	14	45
Grandparent	1	3	**Child's Age at Diagnosis (years)**		
Aunt/Uncle	1	3	0–5	22	71
Great Aunt/Uncle	1	3	6–10	9	29
Brother	1	3	11–14	0	0
Teacher	1	3	Children Experiencing Partial Disclosure Before Full Disclosure	13	42

^a^ Percentages may not total 100% due to rounding.

### Challenges Reported by Caregivers of HIV Positive Children

**Fig 1:**
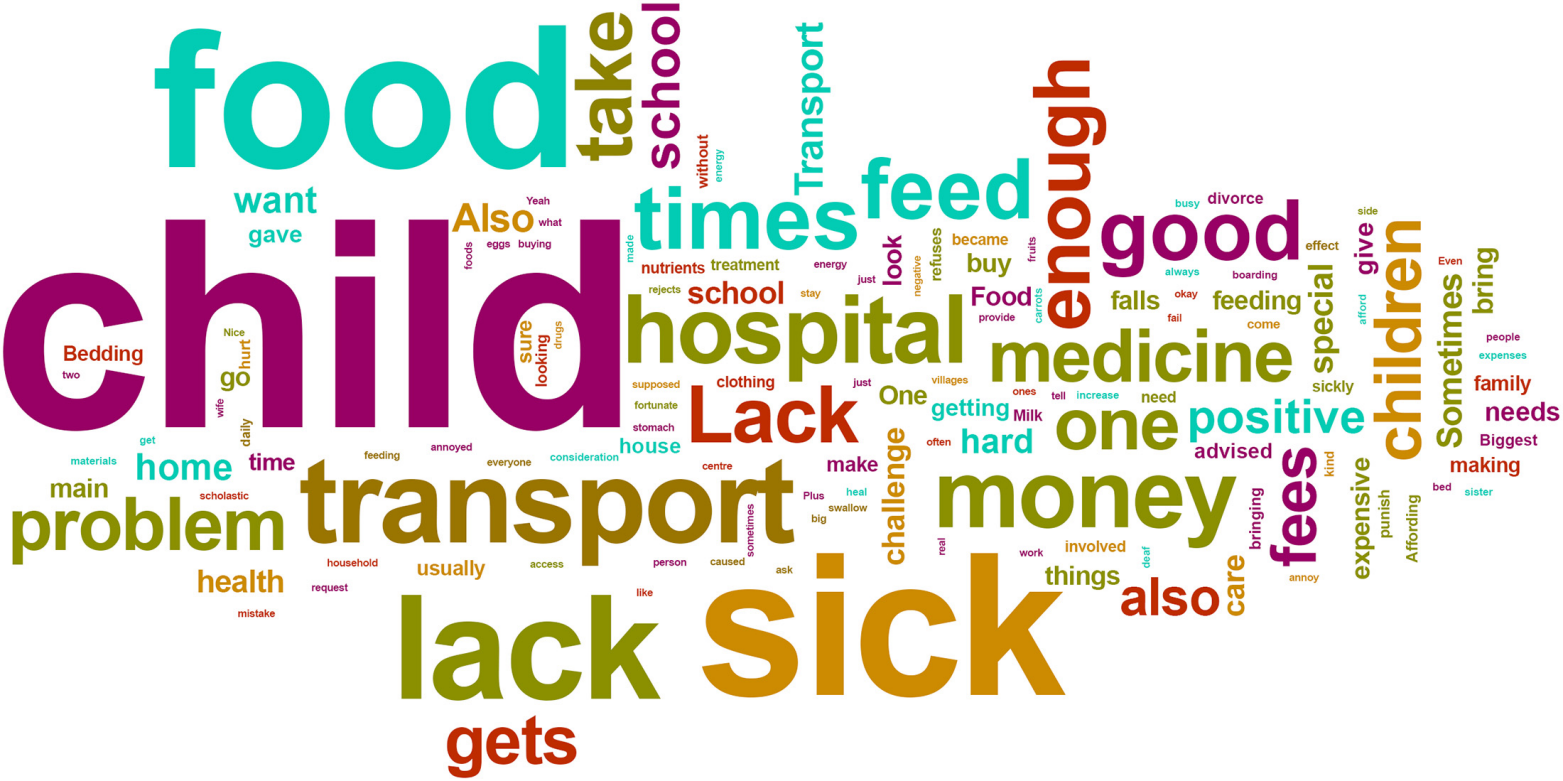
Word Cloud [created using Wordle (www.wordle.net)]. Caregivers’ responses to the statement, “Describe the challenges you face as the caregiver of an HIV-positive child”.

When participants were asked to describe in their own words the challenges they face as caregivers of HIV positive children, several themes were identified by the frequency of specific words that were used in the responses (Fig 1). Caregivers most commonly mentioned difficulty in affording transportation, which was described as being a barrier to keeping appointments at the hospital. Difficulty in affording nutritious food for the child was another common challenge reported by caregivers. Finally, many caregivers reported difficulty in caring for the positive child when the child had fallen ill.

Challenges directly related to the child’s HIV-related medications were also mentioned in the caregivers’ responses, but much less frequently than those dealing with transport, food security and illness. Such challenges included with the child’s medication adherence, lapses in medication availability, and concerns over the side effects of the child’s medication.

### Attitudes and Behaviours Related to HIV Testing

Although difficulty affording transportation was the most common challenge reported by the caregivers (Fig 1), this did not appear to be a significant roadblock to HIV testing or counselling; a majority of all caregivers interviewed considered it relatively easy to access HIV testing and counselling services in their community. Of caregivers who were themselves tested for HIV, a majority chose to do so in response to existing illness either in themselves, their spouse, or the child under their care (Table 2). Similarly, the reasons cited by caregivers for getting the child tested for HIV were overwhelmingly related to the child, the parent, or another family member exhibiting signs of illness (Table 3) with vast majority of children being tested as a response to symptoms of illness in themselves or others. The age at which children were tested for HIV varied greatly (Table 4) and no children were reported to have been tested as part of standard postnatal care (Table 3). Among interviewed caregivers, most reported having the child tested for HIV early in the child’s life, with a majority disclosing the child’s status by the age of 7 (Table 4).

**Table 2:** Reason for testing self for HIV.

Reason	Frequency^a^	%^b^
Encouraged by community leaders to get tested	1	3
Tested as part of antenatal care	3	10
Child was sick/weak	2	7
Spouse got sick or died	5	17
Felt sick/weak	10	34
Just wanted to know HIV status	5	17
Child tested positive for HIV	2	7
Wanted to know if sexual partner was HIV positive	1	3

^a^ Frequency may not total n = 28 due to responses in multiple categories.

^b^ Percentages may not total 100% due to rounding.

**Table 3:** Reason for testing child for HIV.

Reason	Number of Caregivers Reporting this Reason^a^	% of Caregivers Reporting this Reason^b^
Child was sick/weak	23	74
Parent was sick or died	6	19
Sibling was sick/weak	1	3
Parent was known to be positive	3	10
Recommended by health care worker	1	3
Caregiver wanted to know status of child's father	1	3
Tested as part of standard postnatal care	0	0

^a^ May not total n = 31 due to responses in multiple categories.

^b^ Percentages may not total 100% due to rounding.

**Table 4:** Age in Years of Key Events in Disclosure Process.

Event	Mean (Years)	SD (Years)
HIV Testing	4	3
Initiation of HIV Education/Information Process	7	2
Partial Disclosure of HIV Status	6	2
Full Disclosure of HIV Status	7	2
Interval Between Testing and Disclosure	3	3
Interval Between Initiation of HIV Information and Disclosure	<1	<1
Interval Between Partial and Full Disclosure	3	2

### Attitudes and Behaviours Related to Informing Children about HIV

The data showed that the process to begin informing on HIV was initiated at a mean age of 7 years old (Table 4). When the HIV information process was described by the caregivers, the caregivers identified themselves as being most responsible for informing the child about HIV, either by themselves or in concert with an HIV counsellor, in nearly all cases (Table 5).

**Table 5:** Person Most Responsible for Providing HIV Information.

Relationship	Frequency	Percent
Caregiver Alone	25	84%
Counsellor Alone	1	3%
Caregiver and Counsellor Together	3	10%
Other Relative	1	3%

When asked to describe the process by which the children were informed about HIV, the caregivers’ responses revealed several themes. First, many caregivers expressed doubt that the child understood what they were being told, especially when the child learning about HIV was below the age of 7 (Box 1).

**Box 1. Selected responses illustrating emerging theme of doubts that the child truly understood when informed of their HIV status.**

“The first time [we disclosed] I wasn’t sure if she understood. Now I know she knows.” (Biological mother, 38 years old, HIV positive, Status disclosed at age 5).

“She asks me every time, like every two months she will ask me why she has to swallow the medicines.” (Biological mother, 29 years old, HIV positive, Status disclosed at age 7).

“He didn’t react [when told]. I didn’t notice a change, as if he didn’t understand.” (Biological father, 44 years old, HIV positive, Status disclosed at age 5).

“He didn’t react [when told] … He is too young to know.” (Biological mother, 50 years old, HIV positive, Status disclosed at age 7).

**Fig 2:**
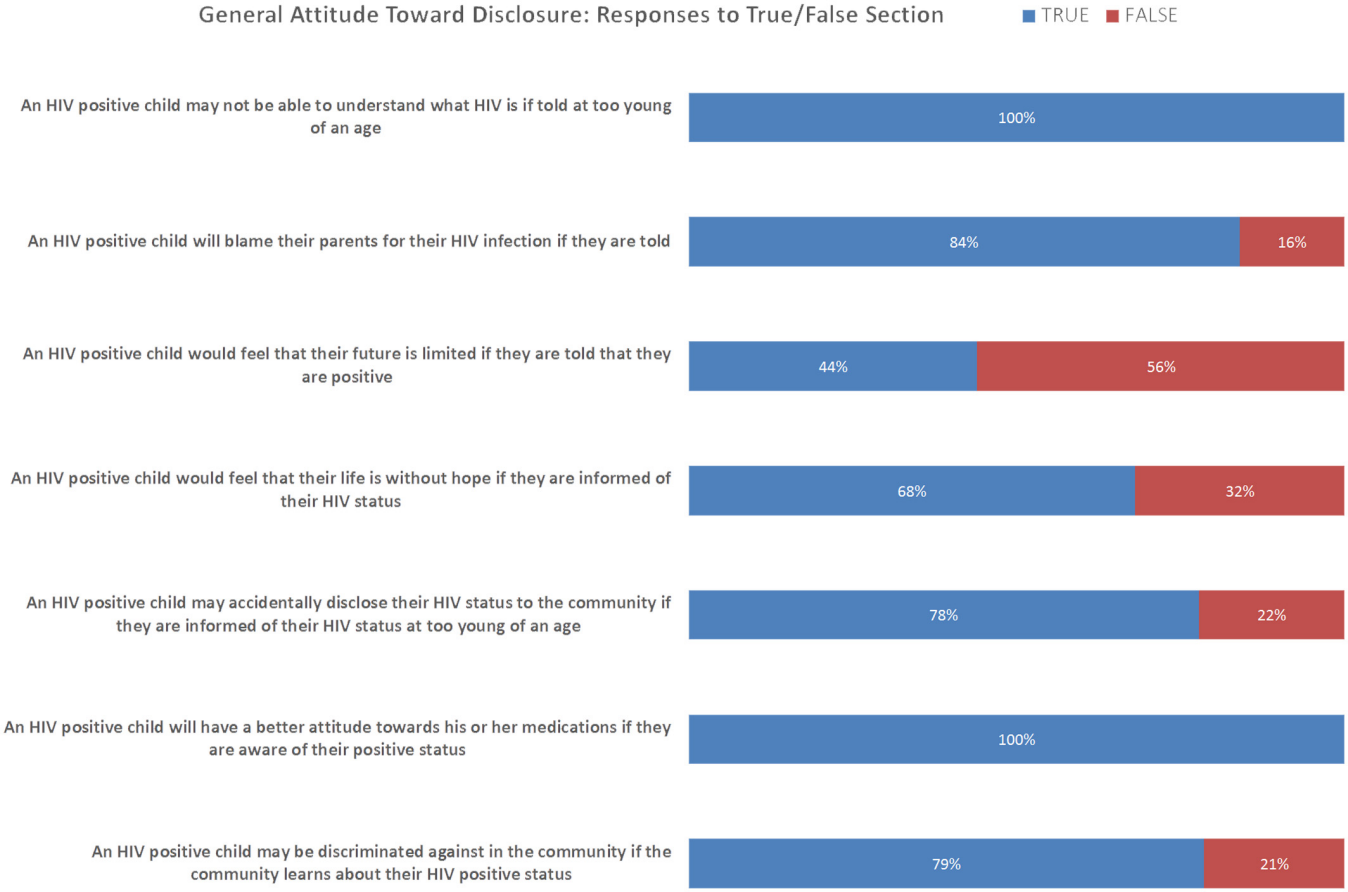
General Attitude to Disclosure.

A lack of reaction on the part of the child was another sign caregivers interpreted to mean when explaining their feelings that the child did not understand (Box 1). Other caregivers reported that the child felt, or might feel that their diagnosis was a death sentence, with 68% believing that a child would feel that their life was without hope if they knew their status (Box 2, Fig 2).

**Box 2. Selected responses given when asked to expand on True/False answer to statement: “An HIV-positive child will feel that their life is without hope if informed of their HIV status”**

**Caregivers who said “True” (68%):**

“Because after her elder [10 year old] sister had learned that [her younger sister] was HIV positive, she told the grandmother that she should poison [her] so she could die, because there is no future.”

(Biological mother, 29 years old, HIV positive, Status disclosed at age 7).

“From the moment you tell the child they have AIDS, I think they will lose hope, because you will think you will die any time. Later on, they may understand that the ARVs [antiretroviral medications] will help them live longer.” (Brother, 19 years old, HIV status unknown, Status disclosed at age 8).

“One child I knew refused to swallow his ARVs because he felt he didn’t have a future.”

(Biological mother, 30 years old, HIV positive, Status disclosed at age 6).

“True, because they don’t really know what AIDS is. They don’t know that with ARVs they can still live longer.” (Uncle, 56 years old, HIV negative, Status disclosed at age 7).

“True, because they think that at any time they are going to die.” (Biological mother, 38 years old, HIV positive, Status disclosed at age 5).

**Caregivers who said “False” (32%):**

“Because if they informed him or talked to him more about HIV he won’t feel that way.”

(Biological father, 45 years old, HIV negative, Status disclosed at age 6).

“If you tell the truth, and tell her that she will live longer with medication she won’t lose hope.” (Biological mother, 36 years old, HIV positive, Status disclosed at age 6).

“…if you tell them when they are young they will think that HIV is normal, since they are growing up like [other children].” (Biological mother, 36 years old, HIV positive, Status disclosed at age 6).

“If the child thinks that they can be cured, they won’t lose hope.” (Stepmother, 24 years old, HIV positive, Status disclosed at age 3).

### Attitudes and Behaviours Related to Disclosure of HIV Status

Following initial open-ended questions, caregivers were read a brief section of statements and asked whether they felt the statements were mostly true or mostly false (Fig 2). The purpose of this section was to act as a comparison to open-ended responses recorded earlier in the interview as well as to prompt additional open-ended questions.

Many attitudes and expectations related to HIV disclosure were shared by the caregivers interviewed. All caregivers felt that a child who knows his or her HIV status will have a “Good attitude towards his or her medications”. However, a significant majority of caregivers relayed that a child with HIV would blame their parents for their HIV infection if their status were disclosed (Box 3), and many reported that children who are HIV positive are discriminated against in their community (Box 4, Fig 2).

**Box 3. Selected responses given when asked to expand on True/False answer to statement: “An HIV-positive child will blame their parents for their HIV infection if they are told.”**

**Caregivers who said “True” (84%):**

“…those who got the AIDS from their parents will blame them because they are the ones who infected them.” (Biological parent, 30 years old, HIV positive, Status disclosed at age 5).

“Because they feel that they are innocent, and if the mother didn’t have AIDS they would be free.” (Biological mother, 40 years old, HIV positive, Status disclosed at age 7).

“I think the child would think that, if my mom didn’t have AIDS, [the child] would be free.”

(Biological mother, 36 years old, HIV positive, Status disclosed at age 7).

“They would blame the parents because the child is innocent, and it’s the parents who gave them the disease.” (Uncle, 56 years old, HIV negative, Status disclosed at age 7).

**Caregivers who said “False” (16%):**

“False, because if they have taught him and shown him how HIV is spread, they won’t blame it on the parents.” (Biological father, 45 years old, HIV negative, Status disclosed at age 6).

**Box 4. Selected responses given when asked to expand on True/False answer to statement: “An HIV-positive child will be discriminated against in your community if the community learns of the child’s HIV status.”**

**Caregivers who said “True” (79%):**

“…the majority are not informed about AIDS. They tend to think that, if [the child] goes with them, he will spread the disease.” (Biological father, 45 years old, HIV negative, Status disclosed at age 6).

“They may have misconceptions, like that AIDS is spread through sharing food. They may not want the children to play together.” (Biological mother, 36 years old, HIV positive, Status disclosed at age 7).

“Our relatives and community members were telling her that she was already dead, that she has no use.” (Biological mother, 38 years old, HIV positive, Status disclosed at age 5).

“True, because I see her cousins pinpoint her and refuse to eat with her.” (Biological mother, 29 years old, HIV positive, Status disclosed at age 7).

“When they know you are positive, they tend to push you away, children especially.” (Grandfather, 73 years old, HIV negative, Status disclosed at age 8).

“Yes. Some people tend to think that, when the child eats food with other children who are HIV negative, they think the HIV would spread. Or, when they play games, [the child] could hurt themselves and spread HIV.” (Biological mother, 40 years old, HIV positive, Status disclosed at age 7).

“True, because they think that they will spread the HIV to others.” (Biological mother, 38 years old, HIV positive, Status disclosed at age 11).

“They think the child will spread the sickness to their children.” (Brother, 19 years old, HIV status unknown, Status disclosed at age 8).

“Because they will think they will spread the disease to the children who are negative.” (Biological mother, 39 years old, HIV positive, Status disclosed at age 9).

**Caregivers who said “False” (21%):**

“False, because at the time many people are infected with HIV.” (Biological mother, 30 years old, HIV positive, Status disclosed at age 5).

“…people know how HIV is [sic] and they will take it as normal.” (Biological mother, 43 years old, HIV positive, Status disclosed at age 13).

Nearly half (42%) of caregivers admitted to initially telling the child that they were sick with a disease other than HIV (Table 1), with malaria being the most common disease mentioned in this way. This partial disclosure occurred at a mean age of 6 years (Table 4). When prompted to discuss their motivations for deceiving the child, a theme of feeling that the child was too young or otherwise unable to understand emerged (Box 1). Among other caregivers who chose to partially disclose the illness to the child, many expressed fears about how the child will react to the knowledge.

Full disclosure of HIV status, when it occurs, typically happens at age 7 (Table 4). Similar to the pattern observed with respect to who is most responsible for providing the child information on HIV, the caregiver interviewed identified themselves as the person who disclosed the status to the child in the vast majority of cases (Table 6). The amount of time between when the child first began to be informed about HIV and the initial disclosure event was less than a year in the vast majority of cases (Table 4).

**Table 6:** Person who disclosed HIV status to child.

Person	Frequency	%
Caregiver	23	82
Other relative	2	7
Doctor	2	7
HIV counsellor	1	4

When asked to describe the process by which caregivers prepared themselves and the child prior to the initial disclosure event, two main yet contrasting themes were evident. Responses commonly described seeking out an HIV counsellor or health care professional for advice on what they should say or do when disclosing. When pressed for additional details as to the type of instruction these information sources provided, caregivers were unable to provide specifics. The other theme common to many responses was a lack of preparation of any kind before initiating disclosure. In either case, the decision to disclose was made in response to the child asking questions about his or her medication or refusing to either take the medication or attend regular clinic appointments (Box 5).

**Box 5. Selected responses to question: “What made you decide to tell the child that he or she had HIV?”**

**Caregivers who mentioned medication adherence as main or sole reason:**

“Because she asked why she was taking her medicine.” (Biological mother, 30 years old, HIV positive, Status disclosed at age 5).

“I wanted him to not refuse to take his medicine.” (Biological mother, 36 years old, HIV positive, Status disclosed at age 6)

“I was asked why she was swallowing the medicine, and I told her the truth.” (Biological mother, 36 years old, HIV positive, Status disclosed at age 7).

“[she] had been refusing medicines, and the hospital advised us to tell the child” (Grandfather, 73 years old, HIV negative, Status disclosed at age 8).

“We wanted him to know so he would take his medicines well and on time” (Biological father, 44 years old, HIV positive, Status disclosed at age 5).

“She was asking why she had to swallow the medicine.” (Biological mother, 36 years old, HIV positive, Status disclosed at age 6).

“I saw that she was mature, and she was asking why she was taking the medicines.” (Biological mother, 38 years old, HIV positive, Status disclosed at age 10).

When asked to describe what they said during the initial disclosure event, HIV positive caregivers commonly reported disclosing their own HIV status to the child at the same time they disclosed the child’s. Similarly, caregivers who were not the biological parent of the child commonly reported informing the child that his or her parent was also HIV positive as part of the initial disclosure event. Caregivers also described stressing to the child that taking their HIV-related medications was crucial to their health and future, although many also reported telling the children that their antiretroviral medications would eventually cure them of the disease (Box 6).

**Box 6. Selected responses to question: “Please tell me, in your own words, what you said [when you disclosed the status to the child]?”**

“I told her that I was sick, and that when I gave birth to you, you were sick. But don’t worry, we will both swallow the medicine and we will be fine. It will make us both good. In time, we won’t need to swallow the medicine because we will be healed.” (Biological mother, 39 years old, HIV positive, Status disclosed at age 9).

“He [the doctor] told him that he was tested and found HIV positive, but that I am going to give you this medicine to take and with time you’ll be okay.” (Stepmother, 24 years old, HIV positive, Status disclosed at age 5).

“I told him, if you don’t swallow the medicine, you are going to become very sick like the way you were before. Also, I told him that he got AIDS when I was giving birth to him.” (Biological mother, 36 years old, HIV positive, Status disclosed at age 6).

“I told him that the father spread the HIV to me and he was born with HIV. I also warned him that he needs to take his medicine regularly.” (Biological mother, 30 years old, HIV positive, Status disclosed at age 6).

“I told her to fear AIDS and I told her how it is spread. Then I told her that she got it from us, the parents. Finally, that she should take the drugs and never spread it to others.” (Biological mother, 38 years old, HIV positive, Status disclosed at age 10).

“I told him that you are HIV positive and you are to be on this medication for the rest of your life.” Biological mother, 43 years old, HIV positive, Status disclosed at age 13).

“I explained to her that she and I were both sick, and that she should learn to love herself the way she is. Also left her under the care of her godmother if I wasn’t there anymore.” (Biological mother, 36 years old, HIV positive, Status disclosed at age 7).

“When she was leaving for boarding school, I sat her down and told her to take her medicine. I also told her to not share any sharp instruments with your friends because you could spread HIV. She asked me, ‘I have HIV?’ and I said ‘Yes, you got it when I was producing you”. (Biological mother, 38 years old, HIV positive, Status disclosed at age 5).

“I told her that your father recently passed away. He died of AIDS. Also, you are born with AIDS. You need to take your medicine, it will help you to live longer.” (Biological mother, 29 years old, HIV positive, Status disclosed at age 7).

“[when leaving hospital] I asked him ‘Do you know where we are coming from, and what we were doing there?’ He said no. I told him, ‘You are there to collect this medicine. This medicine is for HIV, and you are HIV positive. And the HIV, you got it from your parents.” (Uncle, 56 years old, HIV negative, Status disclosed at age 7).

“We told him that he had HIV, that he got it from both his parents from birth when his mother was producing him. We told him about what his medicines do, how the ARVs help him.” (Biological father, 44 years old, HIV positive, Status disclosed at age 5).

### Perceived Effects of HIV Status Disclosure

Of those caregivers who described the experience of disclosing HIV status to an HIV-positive child under their care, 80% believed that the way in which they did so was a “good idea” and that disclosing the status to the child resulted in positive outcomes (Box 7). However, an important theme emerged following disclosure was the doubt expressed by the caregiver whether the child understood what they were being told. This theme recurred in the accounts of multiple caregivers and across the full range of ages of disclosure (Box 1).

**Box 7. Selected responses to the question: “Do you think that telling the child when you did and how you did was the right decision?”**

**Caregivers who said “Yes” (80%):**

“At that age, the child was ready and could understand what it meant [to have HIV].”

(Biological mother, Age 30, HIV positive, Status disclosed at age 5)

“I wanted her to know in case I die that she has AIDS, and so she wouldn’t learn it from other people.” (Biological mother, Age 40, HIV positive, Status disclosed at age 7)

“If I didn’t tell him, he would still be refusing to swallow his medicine.” (Stepmother, Age 24, HIV positive, Status disclosed at age 3).

“Yes, he is determined to swallow his medicine.” (Stepmother, Age 24, HIV positive, Status disclosed at age 5).

“I wanted him to know, so he wouldn’t be asking so many questions about why he was swallowing the medicine.” (Biological mother, Age 36, HIV positive, Status disclosed at age 6).

“I wanted her to be comfortable with her life, not compare herself to everyone who doesn’t have it, and also not to blame me because she got AIDS through me.” (Biological mother, Age 36, HIV positive, Status disclosed at age 7).

**Caregivers who said “No” (20%):**

“She wanted to hang herself. She went down to this river nearby to drown herself. We stopped her on the way there… I took her to the hospital to close her in a room for a week so she wouldn’t hurt herself.” (Stepmother, Age 38, HIV positive, Status disclosed at age 9).

“The child started hating herself after I told her she had AIDS. Also, at that age I don’t think she was ready to hear it.” (Biological mother, Age 29, HIV positive, Status disclosed at age 5).

“No, because the child wasn’t old enough at the time … I should have told him at around 7 years.” (Biological father, Age 45, HIV negative, Status disclosed at age 5).

### Triangulation of Results Within Methodology

Caregiver responses tended to remain consistent throughout individual interviews. However, three notable exceptions are described below.

When asked to tell the interviewer a story about a time when the child under their care missed a dose of ARVs or antibiotics, 39% said the child never missed a single dose. Within this subgroup, however, responses to open-ended questions revealed an emergent theme of difficulties in medication compliance Medication compliance was reported to either be improved following HIV status disclosure, or as a result of the child being informed of their HIV status because they were refusing to take their medication. This inconsistency may suggest that a proportion of caregivers may be reluctant to openly admit instances of medication noncompliance, a phenomenon that has been previously observed in multiple studies [10, 30]. Alternatively, the apparent discrepancy between results could reflect that children were reluctant to take medications pre-disclosure, and that this reluctance was diminished following disclosure.

Another discrepancy occurred between caregiver response to the statements “An HIV-positive child will feel that their life is without hope if informed of their HIV status” and “An HIV positive child will feel that their future is limited if informed of their HIV status.” (Fig 2) While 68% responded true to the first statement and 44% responded true to the second statement, only 68% of these caregivers responded to both statements in the same way. Upon further discussion, it became clear that the caregivers were interpreting the term “future” to refer to the child’s prospects of obtaining an education or employment.

Finally, caregivers’ general attitudes toward disclosure were surprisingly negative, with caregivers agreeing that children who learn their positive status may be discriminated against in their community, feel their life is without hope, or blame their biological parents for their infection (Fig 2). These attitudes were present among caregivers despite 80% of them believing that disclosing HIV diagnosis to children under their care was the right decision (Box 7). This may be due to the caregivers observing the negative outcomes experienced by other members of their community during their own disclosure experiences, a rationale that was mentioned by several caregivers.

## Discussion

When looking at the disclosure experiences of caregivers from start to finish, several key differences from current guidelines become apparent. Current recommendations for disclosing a child’s HIV status include the formation of a disclosure plan between the caregiver and an HIV counsellor that takes into account the family’s dynamics, the child’s intellectual development, and the goals of the caregiver. Once the plan is in place, the child should be informed about the disease in a developmentally appropriate manner prior to the initial disclosure event. Once informed of their HIV status, guidelines suggest that the child and caregiver continue to discuss the child’s HIV status and its impact on their life in growing complexity as the child matures intellectually [21–23]. However, the caregivers’ responses indicated that more than a third (36%) of the respondents embarked on disclosing the status to the child without first seeking help. Many more caregivers expressed doubts that the child had indeed understood what they were being told. This is supported by recent research involving caregivers of HIV-positive children in Mbarara, Uganda as well as in the Democratic Republic of Congo, both of which suggest that caregivers tend to view HIV status disclosure as a single step as opposed to an ongoing process [25, 26]. This lack of involvement on the part of health care workers and HIV counsellors in the pre-disclosure preparation may lead to sub-optimal or harmful disclosure experiences.

The narratives provided by the caregivers looked at who they sought out for advice regarding disclosing the HIV status to the child and what they did to prepare themselves and the child before the initial event. None of these narratives touched upon whether these choices were made against the advice of their health care providers. Further, when caregivers were able to describe the nature of advice provided to them by HIV counsellors or other health care workers, none of these descriptions included expected points such as approaching disclosure in a stepwise manner, the formation of a disclosure plan, or the counsellor acting as a facilitator for the HIV information and disclosure process.

In nearly all cases, it was the caregiver or another relative who disclosed the HIV diagnosis to the child. Only rarely, in 11% of cases (Table 6), was the HIV status initially disclosed by a health care worker such as a physician or HIV counsellor. This is in line with data from multiple other studies conducted throughout Sub-Saharan Africa [15, 26]. Current guidelines do not specify who should disclose the diagnosis to an HIV-positive child, be it caregiver, counsellor or health care professional, so long as that person is selected while being mindful of the effect of HIV status disclosure on the child’s well-being [23].

The data collected here suggest that, regardless of the age of disclosure, provision of HIV information tends to begin immediately prior. Thus, children with little or no HIV knowledge may be inundated with information in a relatively short period of time, culminating in the disclosure of their HIV status. This occurred over a wide range of intervals (Mean = 3 years, SD = 3 years) between the child testing positive and the initial disclosure event (Table 4). Learning their HIV status in this manner, as opposed to a more stepwise approach that tailors the information given to the child’s intellectual development, may lessen the positive effects of disclosure.

The rate of partial disclosure reported by interviewees revealed that nearly half of all positive children were first only told that they were sick, or were told that they had a disease other than HIV. Further, among children who experienced partial disclosure prior to full disclosure, this deception would typically last for several years. This practice reflects the caregivers’ reported doubts that the children were capable of understanding full disclosure of their HIV status, but the duration of partial disclosure may also suggest a reluctance to fully disclose that was not explicitly addressed in the interview responses. In either case, the frequency and duration of partial disclosure represent an aspect of the disclosure experience that may be a target for future interventions.

Across the entire range of disclosure ages, there was a persistent theme of caregivers doubting that the children fully understood the information shared at the time of disclosure. This may be due to baseline differences in the quality or extent of instruction the child and caregiver received prior to beginning the disclosure process, something that was not controlled for in this study. Another possible explanation is that the malnutrition common in HIV positive children may result in delays in cognitive development., Up to 50% of children initiated on ARV therapy in African HIV treatment programs are undernourished [33]. Severe malnutrition, sufficient to cause stunted growth, is linked to structural and functional damage to the brain, which may delay cognitive development [34]. Even in situations where malnutrition is not severe, early nutritional intake is a predictor of later cognitive ability [35, 36]. For these children, the standard recommendation of disclosure at ‘school age’ may result in disclosure too early for them to reasonably understand. To date, the existence of a link between malnutrition and poor HIV disclosure outcomes has not been investigated and thus provides the opportunity for future study. If a link were found it could mean that, for this patient population, the optimal ages to begin the HIV education process and disclosure process may differ from those recommended by current guidelines.

Many caregivers responded in the affirmative to the statement that a child would feel that their life is without hope if they learned of their status (Fig 2). The reported reactions of some of the children who learned their status, which ranged from confusion to stoic acceptance to self-harm or attempted suicide, lends credence to this. As access to counselling does not appear to be a significant roadblock, educating caregivers and counsellors in the steps to take prior to disclosure (e.g., planning how to disclose with a counsellor and ensuring that the child has a baseline knowledge of HIV including the knowledge that the disease should not be considered an automatic death sentence) may be pertinent steps to change the disclosure experiences of Ugandan families that may result in more positive outcomes for at-risk children. A longer-term study, where positive children are followed through the disclosure process and beyond, would be the logical next step to identify potential benefits of a more formalized disclosure process that stresses education of caregiver and child. Identifying the baseline instruction health care providers in the area receive regarding HIV disclosure to pediatric patients would be a key first step to completing such a study. Further, assessing the children’s HIV knowledge directly throughout the education and disclosure process would provide more robust data on the child’s true disclosure experience.

Caregivers’ attitudes towards HIV status disclosure were frequently negative, with the overwhelming majority believing that the child would feel that their life was without hope when they learned their status, that they will blame their parents for their HIV infection if told, and/or that they would accidentally disclose his or her status to the community (Fig 2). Interestingly, even caregivers who reported a positive disclosure experience shared the beliefs that disclosure would have negative consequences. Despite these admissions, all (100%) of caregivers believed that disclosure would result in the child having a better attitude toward their medications.

In spite of efforts to promote HIV testing as part of routine medical care, the vast majority of HIV tests discussed in the interviews were motivated by active symptoms—either in a child, parent, sibling, spouse, or the individual themselves. The fact that nearly every respondent viewed HIV testing and counselling services as easy to obtain in their community suggests that more education and outreach may be necessary to convince at-risk individuals in rural Uganda to get tested for HIV before they fall ill. Such efforts would also serve to address the fact that the vast majority of interviewees report that, in their communities, HIV-positive children would be discriminated against if their status becomes known (Fig 2).

The study suffers from several limitations. Although the sample size of 28 caregivers discussing the experiences of 31 children is more modest than some contemporary studies examining HIV status disclosure in Sub-Saharan Africa, this is largely due to the decision to restrict participation in the study to caregivers who had fully disclosed the HIV diagnosis to the child under their care. As recent evidence in the area suggests [15], this decision would exclude 69% of caregivers presenting at the ISS clinic. Further, the sample size used is significantly larger than many relevant qualitative studies recently released [9, 26].

Another potential limitation involves the challenge inherent in conducting semi-structured interviews via interpreters. Although all translators employed in the study were extensively trained and urged to strive for consistency between all interviews, using mock interviews to identify and address potential issues before they were encountered, some caregivers clearly interpreted certain questions differently than others. To avoid steering interviewee responses to the researchers’ preconceptions or putting words in the subjects’ mouths, the interviewees were simply requested to clarify their position before responses were coded ‘as is’. By using a second translator to validate the first translation, we feel that all questions and answers are recorded as accurately as possible using this method.

The original study design was going to compare the responses of caregivers recruited at the ISS clinic with those recruited through FAOC. As we began to collect data, however, we noticed that there was significant overlap between the two groups. For one thing, caregivers recruited via FAOC were often receiving their treatment in Mbarara and caregivers recruited at the ISS clinic often were living in the rural parishes surrounding Mbarara. Thus, the rural vs. municipal comparison was not possible, as the two methods of sampling were drawing participants from the same populations.

The recruitment process was designed so that neither the interviewers, health care workers, nor FAOC chairpersons would be able to influence who would be invited to participate. Hence that decision to pool the data from the two sampling methods would not introduce bias. In the ISS clinic, all caregivers presenting were screened and, if found to be eligible, all were invited to participate. At FAOC meetings, all members were informed of the study at meetings and were encouraged to contact us if they felt that they met the criteria and were interested in participating. The only potential difference in the two groups would be that the caregivers approached directly at the ISS clinic would be more likely to participate than those who were simply made aware of the study at FAOC meetings and had to take the additional step of contacting us to volunteer. We noticed greater numbers of participants volunteering through the ISS clinic vs. the FAOC meetings and thus directly comparing the two groups may not necessarily be valid.

The ethical challenges inherent in interviewing HIV positive children with potentially different levels of awareness of their HIV status prevented the researchers from directly assessing each child’s level of HIV knowledge. It would be unacceptable, for example, for an interviewer to accidentally disclose the child’s HIV status to him or her if the caregiver was somehow misinformed as to the child’s knowledge of their own HIV status. For these reasons, the study was less concerned with the exact knowledge possessed by the children as opposed to understanding when the process of informing began for each child with respect to HIV, and how this fit into the disclosure process. The downside to this focus is that the study is dependent on caregivers being able to accurately assess the extent to which a child understood when they were informed of their HIV status. For this reason, it is possible that the number of children who do not fully understand the realities of their HIV infection following disclosure differs from what caregivers reported. Directly assessing the HIV knowledge of the children involved and how that knowledge changes throughout a stepwise disclosure process will be a crucial component of future research into this topic.

Interview format is, historically, an unreliable way to accurately assess medication adherence, as it introduces both recall and social desirability bias, often resulting in patients over-reporting adherence [37, 38]. For this reason, the study does not investigate potential relationships between a child’s disclosure experience and their drug compliance. Ultimately, determining any effect of disclosure method on drug compliance will be important, especially once the child in question reaches an age where they take responsibility for administering their own medications. In such a scenario, review of medication refill timings and trends in CD4 counts will likely produce the most accurate picture of overall medication compliance.

Finally, many of the questions depend on the recall of the caregiver. As such, the narrative built by each interview to describe the disclosure process and results is subject to the limitations of the caregiver’s memory. To combat this, the questionnaire was designed to avoid influencing what aspects of HIV testing, education, and disclosure the caregiver chose to focus on, instead encouraging the caregivers to speak about issues and experiences that were important to them.

Overall, the discrepancies between the disclosure experiences reported by caregivers and current guidelines may result in sub-optimal outcomes. As access to HIV counselling was not deemed to be a roadblock by all but a few caregivers, changing disclosure practices may be as simple as providing more concrete guidelines for HIV counsellors to follow to ensure that positive children are adequately informed about HIV prior to disclosure. Whether such changes will result in measurably better outcomes is a question that merits further study.
