# Correction: Caregivers' Attitudes towards HIV Testing and Disclosure of HIV Status to At-Risk Children in Rural Uganda

**DOI:** 10.1371/journal.pone.0154169

**Published:** 2016-04-18

**Authors:** The PLOS ONE Staff

There are errors in the author affiliations. The publisher apologizes for the errors. The affiliations should appear as shown here: Rick Lorenz^1^, Eisha Grant^1,2^, Winnie Muyindike^3^, Samuel Maling^3^, Claire Card^4^, Carol Henry^1^, Adil J. Nazarali^1^

**1** College of Pharmacy and Nutrition, University of Saskatchewan, Saskatoon, Saskatchewan, Canada, **2** Ministry of Health, Kampala, Uganda, **3** Faculty of Medicine, Mbarara University of Science and Technology (MUST), Mbarara, Uganda, **4** Western College of Veterinary Medicine, University of Saskatchewan, Saskatoon, Saskatchewan, Canada

## References

[1] Lorenz R, Grant E, Muyindike W, Maling S, Card C, Henry C, et al (2016) Caregivers’ Attitudes towards HIV Testing and Disclosure of HIV Status to At-Risk Children in Rural Uganda. PLoS ONE 11(2): e0148950 doi:10.1371/journal.pone.0148950 26881773 10.1371/journal.pone.0148950 PMC4755560

